# Multifractal Analysis of the Influence of Indole-3-Acetic Acid on Fast-Activating Vacuolar (FV) Channels of *Beta vulgaris* L. Taproot Cells

**DOI:** 10.3390/membranes13040406

**Published:** 2023-04-03

**Authors:** Janusz Miśkiewicz, Zbigniew Burdach, Zenon Trela, Agnieszka Siemieniuk, Waldemar Karcz

**Affiliations:** 1 Institute of Theoretical Physics, University of Wrocław, 50-204 Wrocław, Poland; 2 Physics and Biophysics Department, Wrocław University of Environmental and Life Sciences, 50-375 Wrocław, Poland; 3 Faculty of Natural Sciences, Institute of Biology, Biotechnology and Environmental Protection, University of Silesia in Katowice, 40-032 Katowice, Poland

**Keywords:** FV channel, patch clamp, multifractal analysis, MDFA, time series analysis, long memory effect

## Abstract

In this paper, the multifractal properties of the ion current time series in the fast-activating vacuolar (FV) channels of *Beta vulgaris* L. taproot cells were investigated. These channels are permeable for only monovalent cations and mediate K^+^ at very low concentrations of cytosolic Ca^2+^ and large voltages of either polarity. Using the patch clamp technique, the currents of the FV channels in red beet taproot vacuoles were recorded and analysed by using the multifractal detrended fluctuation analysis (MFDFA) method. The activity of the FV channels depended on the external potential and was sensitive to the auxin. It was also shown that the singularity spectrum of the ion current in the FV channels is non-singular, and the multifractal parameters, i.e., the generalised Hurst exponent and the singularity spectrum, were modified in the presence of IAA. Taking into account the obtained results, it can be suggested that the multifractal properties of fast-activating vacuolar (FV) K^+^ channels, indicating the existence of long-term memory, should be taken into account in the molecular mechanism of the auxin-induced growth of plant cells.

## 1. Introduction

It is well-established that the plant vacuole is a dynamic cellular compartment that can occupy more than 90% of the cell volume and is essential to plant growth and the physiological characteristics of plants [1,2,3]. Potassium (K^+^) constitutes a chief osmoticum, which plays a key role, among others, in regulating turgor and cell expansion [4]. Vacuolar turgor and vacuolar signalling depend on the concerted actions of the tonoplast transporters and channels. The potassium current across the tonoplast is primarily mediated by three main classes of tonoplast nonselective cation channels (NSCCs): the slow-activating (SV), fast-activating (FV) and K^+^-selective vacuolar (VK) channels (reviewed in [5]). SV channels are permeable to monovalent and divalent cations and are activated by cytosol-positive voltages at an elevated cytosolic Ca^2+^ [6,7,8]. In turn, FV channels are permeable for only monovalent cations and mediate K^+^ at very low concentrations of cytosolic Ca^2+^ and large voltages of either polarity [6,9]. Moreover, the FV channels are inhibited by the divalent cations from either side of the vacuolar membrane [10,11,12]. A third type of vacuolar K^+^ (VK) channels is Ca^2+^-activated channels, which are highly K^+^-selective channels [7].

Recently, Burdach et al. [13] showed that auxin (IAA) at symmetrical 100 mM KCl (100 mM KCl in the pipette and the external medium) stimulated an outward current of the fast-activating vacuolar K^+^ channels, which mediated the K^+^ efflux from the cytoplasm into the vacuole but it did not change the K^+^ efflux from the vacuole into the cytoplasm. The single-channel currents, which were recorded in symmetrical KCl, demonstrated that cytosolic auxin changed the open probability of FV channels at positive voltages to a moderate extent, while it significantly increased the amplitude of the single channel outward currents as well as the number of open channels. At the positive voltages, auxin did not change the unitary conductance of the single channels.

In the present study, we addressed the question of whether auxin (indole–3–acetic acid, IAA) modulates the long-term memory (or long-range correlation) of FV channels. The recent results show not only the important role of long-term memory in the activity of ion channels [14,15,16,17] but also draw attention to their non-trivial multifractal characteristics [18,19,20]. Particularly interesting are results presenting the multifractal characteristics of the ion channel current because they show that the opening and closing processes are indeed very complex. What is more, the long memory effects are sensitive to various factors, e.g., it has been shown that the Hurst exponent of SV channels’ dwell time series are sensitive to lead (Met_3_PbCl) presence [16] or multifractal properties of BK (big potassium) channels of Human Glioblastoma Cells depends on the external potential [20]. Within the present study, the influence of IAA, a central player in plant cell growth regulation, on the FV channel’s multifractal properties is investigated to verify the biological significance of the long-memory effects of the ion current.

## 2. Materials and Methods

### 2.1. Plant Material

Red beet (*Beta vulgaris* L.) taproot vacuoles were isolated using the nonenzymatic method that was previously described by Coyaud et al. [21]. Following this method, the vacuoles were directly extruded into a recording chamber by cutting a slice of fresh tissue and rinsing the surface with a bathing solution. The control bath solution in the patch–clamp experiments contained 100 mM KCl, 0.5 mM EDTA, 5 mM MES, 5 mM Tris and 400 mM sorbitol, with pH 7.5 and an osmolality of 650 mOsm. 

### 2.2. Electrophysiological Experiments

All of the electrophysiological experiments were performed in an excised cytosolic side-out patch [22] using an EPC–7 Plus amplifier (List–Medical–Electronic, Darmstadt, Germany). The current and voltage conventions were in accordance with Bertl et al. [23], e.g., the sign of the voltage refers to the cytosolic side, and the positive (outward) currents represent an efflux of cations into a vacuole. The signal was filtered using a five-pole Bessel filter and was recorded on a hard disk at a sampling frequency of 1–100 kHz. The Bessel filter was an integral part of the EPC–7 Plus amplifier. The single channel FV currents in symmetrical 100 mM KCl were elicited via a series of voltages ranging from –140 to +140 mV in 20 mV steps. From a holding potential of –40 mV, where the activity of the FV channels is minimal [9,10], the electrical potential across the tonoplast was changed for 3 s. The pipettes were filled with a solution containing 100 mM of KCl, 5 mM of MES, 5 mM of Tris with pH 6.0, which was adjusted to an osmolality of 580 mOsm with sorbitol. All of the experiments were performed at room temperature (22 ± 1 °C). The patch pipettes were pulled from borosilicate glass tubes (Kimax–51, Kimble Products, Toledo, OH, USA) using a two-stage pipette puller (model L/M–3–PA, List Medical, Darmstadt, Germany), fire-polished using a CPZ 101 microforge (List Medical, Darmstadt, Germany) and coated with Sylgard (Dow Corning, Midland, MI, USA). The patch electrode resistance (for the electrodes that had been filled with the pipette solution) was 2–4 MΩ; the gigaseal resistance was in the range of 5–20 GΩ. The experimental data were stored and elaborated using Patch–Master, Fit–Master software (HEKA Electronic, Lambrecht, Germany) and Dell Statistica (data analysis software system), version 13 (Dell, TX, USA).

### 2.3. Protocol of Auxin Treatment

The effect of 1 µM indole–3–acetic acid (IAA) on the single FV channels was studied. In the experiments, the control bath solution was changed for the new one with the same salt composition, additionally containing auxin (indole–3–acetic acid, IAA) at a final concentration of 1 µM. The solution exchange protocol used in the conducted studies was as follows: the control means the FV single-channel currents recorded in the control medium immediately after gaining cytosolic side-out patches and after every 5 min for 30 min. The current in the presence of IAA was recorded at 0 min (immediately after the control medium was changed for the new one with the same salt composition, additionally containing auxin) and after every 5 min for 30 min. The bath solution in the recording chamber was exchanged within 1 min via the perfusion of the measuring chamber using an SP200 infusion pump (World Precision Instruments, USA).

## 3. Mathematical Background

Fractal properties are observed in many systems: DNA analysis [24,25,26,27], heart rate dynamics [28,29,30], financial time series [31,32,33], meteorology [34,35,36] and many other applications. The most important message relating to systems with fractal properties is the existence of long-range correlations. However, in various analyses, it has been observed that system correlations might depend on the range. Therefore, the extension of the theory was necessary. As a result, multifractal analysis was developed [37,38]. In general, there are two main methods for analysing multifractal time series features: wavelet transform modulus maxima (WTMM) [39,40] and multifractal detrended fluctuation analysis (MFDFA) [41,42,43,44]. Although, the MFDFA method was introduced to analyse correlations in noisy nonstationary time series [41]. For the convenience of the reader, the MFDFA algorithm will be shortly presented here.

The algorithm can be described in the following steps:

1. Let (*x_i_*) denote *i* = 1, …, N equidistant measurements.
2. Then, calculate the integrated signal profile:
  (1) $$Y\left(j\right)=\sum_{\left\{i=1\right\}}^{j}\left(x\left(i\right)-\langle x\;\rangle \right),\;j=1,\;\ldots ,N$$
  where $\langle x\;\rangle =\frac{1}{N}\sum_{\left\{i=1\right\}}^{N}x_{i}\;$
3. If *n* denotes a segment’s length (which will also be referred to as the scale), then the analysed time series can be divided into M_n_ = [N/n] disjoint segments of length *n* (*n* < N). By starting the division independently from the beginning and the end, the 2M_n_ segments are obtained.
4. At each segment *ν*, a local trend should be calculated, i.e., fitted *l*–th order polynomial $P_{\nu }^{\left(l\right)}$ and the trend subtracted from the signal. For a given segment *ν* (*ν* = 1, …, Mn), the variance of the residual series is equal to
  (2) $$F^{2}\left(\nu ,\;n\right)=\frac{1}{n}\sum_{\left\{j=1\right\}}^{n}{\left\{Y\left[\left(\nu -1\right)n+j\right]-P_{\nu }^{\left(l\right)}\left(j\right)\right\}}^{2}$$
5. Finally, the fluctuation function F^2^(*ν*, *n*) is averaged over *ν*’s, and the fluctuation function is calculated for all possible segment lengths, *n*:
  (3) $$F_{q}\left(n\right)=\frac{1}{2M_{n}}{\left(\sum_{\nu =1}^{2M_{n}}{\left[F^{2}\left(\nu ,\;n\right)\right]}^{\frac{q}{2}}\right)}^{1/q}\;,\;q\in \;$$

The main property of F_q_(n) is that for a time series with fractal properties, the fluctuation scales as a power law as a function of the scale:

(4) $$F_{q}\left(n\right)∼n^{H\left(q\right)}$$

Despite the fact that an arbitrary trend function can be used in MFDFA, in practice, the linear trend is used. It is worth stressing that in the case of monofractal H(q) = *const*. Therefore, it does not depend on q. The relationship between the H and q parameters is crucial because it allows magnifying correlations on different scales. The generalised Hurst exponent, H(q), allows for the calculation of the singularity spectrum of the Hölder exponent, f(α) [45]:

(5) $$\alpha \left(q\right)=H\left(q\right)+q\frac{H\left(q\right)}{q},\;f\left(\alpha \right)=q\left[\alpha \left(q\right)-H\left(q\right)\right]+1.$$

α in Equation (5) can be identified with the Hölder index, which is the local measure of function irregularity.

In applications of MFDFA, for a given value of q, function F_q_(n) is calculated for a broad range of scales, n, and the region of linear dependence of ln Fq on ln n is sought. It can be found from Equation (4) that the slope coefficient is, in this case, equal to the generalised Hurst exponent Hq. After repeating the procedures for different q values, the set of exponents H(q) is derived, and consequently, the quantities α and D(α*)* can be obtained.

In practice, there are various computer packages in which MFDAF is implemented, e.g., MATLAB [44], Python [46] or R [47]. Within the presented study, MATLAB-based implementation adapted to Octave was used.

## 4. Results

**Figure 1:**
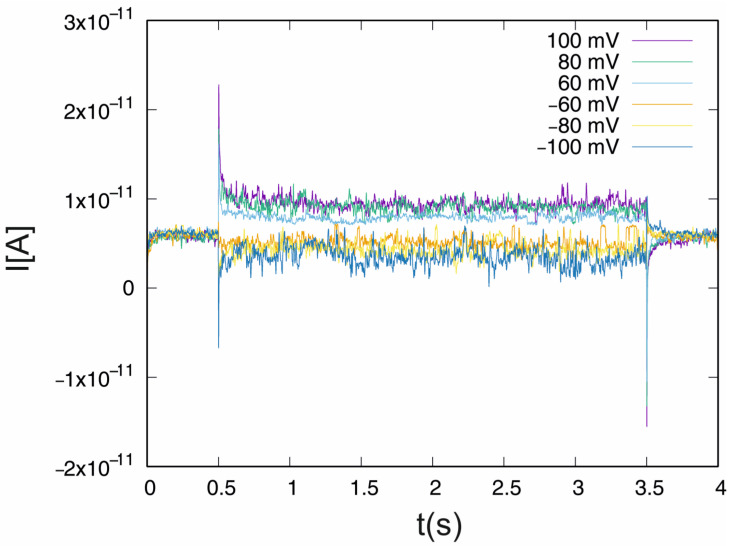
Examples of the signal recordings for the set of external potentials according to the experimental protocol.

The experiment was divided into two parts. In the first, the properties of the ion current under the control conditions were investigated, while in the second part, the influence of IAA at 1 µM was analysed. In the experiment, the ion current time series were recorded using the patch–clamp technique. Each of the recordings consisted of 80,000 data points with a resolution of 5 × 10^−5^ s. According to the experimental protocol, each measurement started at the external potential U *= −*40 mV, which lasted 0.5 s, and then the potential was changed to one with values in the range of U ∈ {−100, −80, −60, 60, 80 and 100 mV}. This potential was maintained for 3.5 s, and the external voltage was returned to the initial value of U = −40 mV. For each of the external potential values, the recordings were repeated five times. Examples of the current recordings (I[A]) performed in the control medium are presented in Figure 1 (one per external potential value).

The applied experimental protocol is reflected in the course of the current records (Figure 1), which show clear fluctuations when the external voltage changes. Due to the presence of current fluctuations triggered by external potential changes, multifractal analysis was applied to the interval from 0.501 s to 3.49 s. Therefore, the analysis is performed on the time series of 59,600 data points.

In the MFDAF analysis, the following functions were calculated and discussed: the generalised Hurst exponent H(q) (which measures how chaotic or unpredictable a time series is) and the singularity spectrum, i.e., f(α) (which quantifies the degree of nonlinearity in the processes). Since each experiment was repeated five times, the mean value and standard deviation of the estimated parameters are calculated and discussed. In the following, the generalised Hurst exponent H(q) will also be denoted as Hq.

### 4.1. Control System

**Figure 2:**
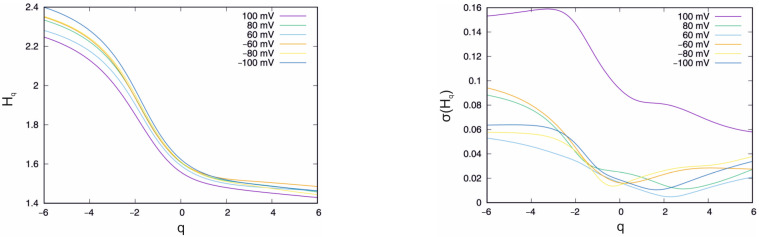
Mean value (**left**) and standard deviation (**right**) of the generalised Hurst exponent Hq.

In the first part of the analysis, the influence of the external voltage on the properties of the single FV channel currents in the control medium was investigated. The mean value and standard deviation of the generalised Hurst exponent as a function of the q parameter for the chosen set of external potentials are presented in Figure 2. It can be observed that the external potential influences the Hq function. This can be seen especially clearly for extreme values of external voltage, i.e., U *=* −100 mV and U = 100 mV. Considering the extreme external potential, the biggest differences are present for the smallest values of q (at q *=* −6, the mean value of Hq differs by ≈ 0.15, while at q = 6, this difference is ≈ 0.035). An analogous effect is seen in the standard deviation plot (Figure 2, right), in which the most significant difference induced by the external potential is observed for the low-value q parameter. Therefore, it can be concluded that the greatest impact of the external potential is observed for the short-range correlations. Very intriguing is the separation of the Hq standard deviation function at U *=* 100 mV. The obvious effect of the external potential is the increase in the dispersion of the generalised Hurst exponents. However, the distance between the curves for the other values of external potentials is too small and does not justify this hypothesis. However, it should be stated that the significance of the shift from the other standard deviation curves indicates nontrivial changes in the system caused by external potential. This effect will be investigated in another study.

**Figure 3:**
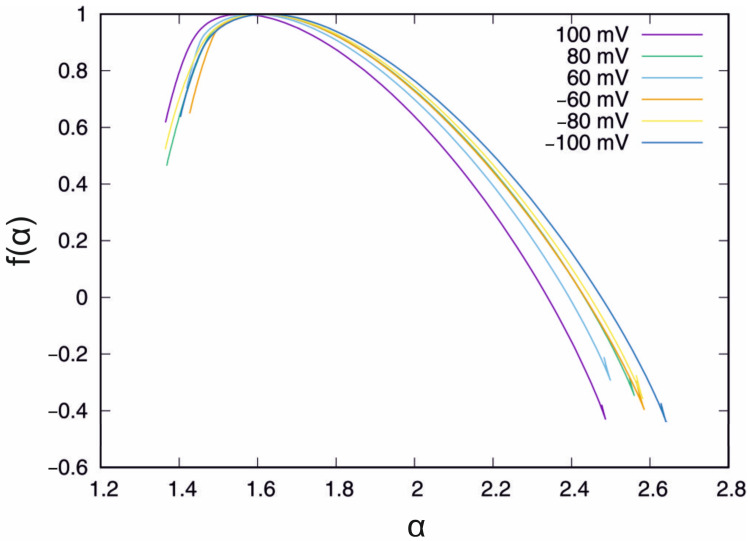
Singularity spectrum *f(α)* obtained for the given set of external potentials.

The second important outcome of the MFDFA analysis is the singularity spectrum presented in Figure 3. The most striking observation is the fact that all the curves in Figure 3 have a strictly multifractal shape (to be compared to other examples, e.g., in [46]). The singularity spectrum f(α) for the monofractal time series or white noise is represented by a single point or very narrow curve due to the limitation of the time series length. Again, as has been observed, multifractal properties depend on external potential. This is particularly seen in the extreme values of an external potential. The singularity spectrum curves corresponding to the potentials of U = −100 mV (navy blue) and U = 100 mV (violet) are the lowest, and the top curves in the plot, Figure 3, for α > 1.6, and is thus above the maximums. In addition to the clear observation that the singularity spectrum is sensitive to external potential, the attempt to define the relationship would be difficult at the present stage of research due to the limited number of experimental data.

The width of the singularity spectrum analysis is presented in Table 1. The biggest mean range of the singularity spectrum is observed at the external potential of U = −100 mV.

Additionally, at this potential, the standard deviation of the received ranges is the smallest. The main conclusion of the ion channels MFDFA analysis at the control solution is that the ion current recordings are multifractal. The second conclusion is that this property is sensitive to external potential. However, the detailed determination of the relationship requires targeted experimental research, which will be undertaken in another study.

### 4.2. Influence of IAA on Multifractal Characteristics

**Figure 4:**
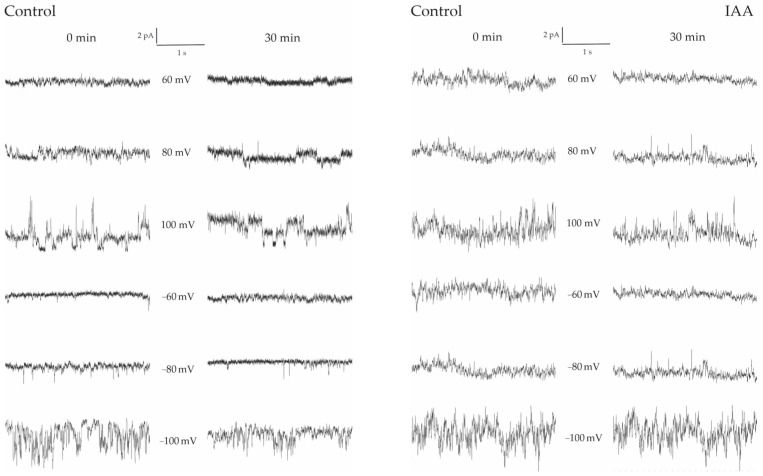
Examples of fast-activating currents in excised vacuolar side-out membrane patches incubated in the control medium and in the presence of auxin (IAA). The currents were measured at symmetrical 100 mM KCl. On the left-hand side, the control at 0 min and 30 min with the same patch is shown. However, on the right-hand side, the control at 0 min and in the presence of auxin (30 min) with the same patch is shown.

The influence of IAA at 1 µM on the ion current of FV channels was investigated according to the following protocol: before the current recordings, the patch was incubated in IAA for one of the periods (0, 5, 10, 15, 20, 25 and 30 min). The period of 0 min means that the current recording was taken immediately after the control medium was changed for the new one with the same salt composition, additionally containing auxin. The results were compared with the data collected in the control solution (Figure 4). 

**Figure 5:**
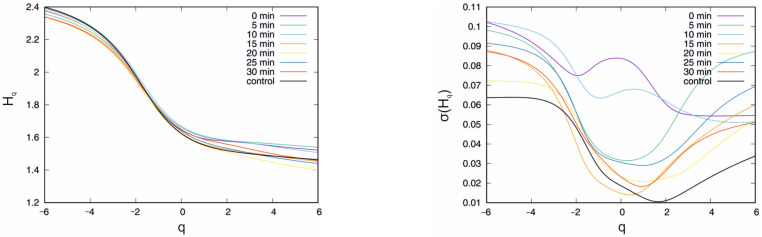
Mean value (**left**) and standard deviation (**right**) of the generalised Hurst exponent Hq in the presence of IAA at the chosen incubation times. U = −100 mV.

**Figure 6:**
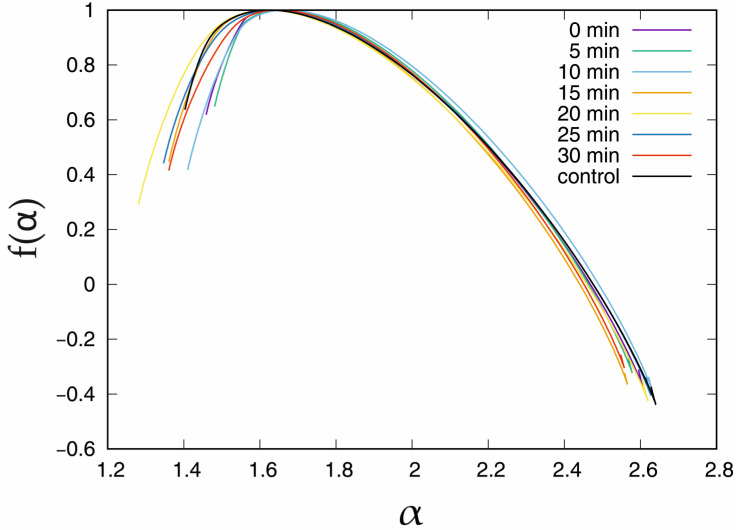
Singularity spectrum f(α) obtained in the presence of IAA for the chosen incubation times. U = −100 mV.

The external potential was set to U = −100 mV. It is worth recalling that, at U = −100 mV, the mean of the singularity spectrum width is the highest while the standard deviation is the lowest of the analysed cases (Table 1). The results of the analysis of the ion current multifractal features are presented in Figure 5 and Figure 6. The mean value curve of the generalised Hurst exponent Hq obtained at the chosen IAA incubation time is presented in Figure 5, left. 

The black curve shows the Hq obtained with the standard solution. The clearest for the interpretation is interval q ∈ (−6, −2), which corresponds to the short correlations in the time series. In this interval, the top curve corresponds to the measurement in the control solution while the bottom corresponds to the longest − 30 min incubation period. However, for positive values of the q > 0 parameter, this observation cannot be sustained. In this case, the Hq obtained for short incubation periods, i.e., 0 min, 5 min and 10 min, are clearly above the control curve (by ≈0.06). The long incubation periods, i.e., 15 min and longer for q > 5, mean that the Hq is below the Hq for the control solution. The bottom curve was observed in the 20 min IAA incubation period. However, it should be stressed that the differences between the mean values of the generalised Hurst exponent in Figure 5 are very small; thus, the present results are rather qualitative than quantitative. The standard deviation of the σ(Hq) is presented in Figure 5, right. In this case, the presence of IAA and the incubation time affect the curves. The black line denoting the time series recorded in the control solution is the lowest curve for small (q < −2) and high (q > 1) values of the q parameter, which means that IAA affects short- and long-range correlations. In the interval q ∈ (−2, 1), the exception is the curve of the 15 min incubation period, which is placed below the standard deviation of the generalised Hurst exponent registered in the control bath. In fact, in the interval *q* ∈ (−2, −1), almost all functions σ(Hq*)*, except for the curves of the incubation periods of 0 min and 10 min, are placed very close to each other. In the case of 0 min and 10 min, besides the local minimum in the interval q ∈ (−2, 2), which is observed for all standard deviation plots, there is also a local maximum − at q = 0 for 0 min and q = 0.8 for 10 min. On the other hand, the standard deviation curve for other incubation periods increases; thus, the local maxima for q > 6 are not excluded. However, the reliable estimation of σ(Hq) for q > 6 requires a longer time series. Considering the information provided by the mean value and the standard deviation, the presented observations are nontrivial. The presence of IAA influences both statistical parameters. In the case of biology, the systems have a natural diversity, and it is observed that the dispersion of the multifractal properties is sensitive to the presence of IAA. The singularity spectrum obtained in the presence of IAA for the given incubation times is presented in Figure 6. The black curve denotes the singularity spectrum for the control solution. The clearest observation is that in the presence of IAA, the general multifractal type of the singularity spectrum holds (Figure 6).

However, the presence of IAA in the solution induces several changes. For the small value of α < 1.6, all of the singularity curves obtained in the presence of IAA differ significantly from the final point. The coordinates of the final point of the singularity spectrum are presented in Table 2.

For the short incubation times, i.e., 0 min and 5 min, the α parameter is greater than that for the outcome of MFDFA in the control bath, while after the longer incubation periods, ≥10 min, the f(α) visually decreases. On the second side of the singularity curves, (α > 1.6), the differences between the singularity spectrum for the control solution and those with the presence of IAA are less noticeable than for (α < 1.6) side. Almost all of the singularity spectrum curves (except 10 min incubation) are located below the control solution singularity spectrum function. Although a detailed discussion of the incubation effect of IAA on the singularity spectrum would be desirable due to the small differences between the individual curves, it is not possible to undertake this discussion due to the biodiversity of the experimental materials. 

Finally, the mean value of the singularity spectrum width and standard deviation were calculated and presented in Table 3.

The presence of IAA in the system within short times (0 min and 5 min) results in the decreased mean width of the singularity spectrum. However, the extension of the incubation time with IAA results in increased spectrum width. The highest value of the mean width was observed for the 20 min incubation time. The effect of the presence of IAA is also visible in the standard deviation of the singularity spectrum width (Table 3). The presence of IAA increases the dispersion of the singularity spectrum by more than two times.

## 5. Discussion

Since then, the patch–clamp technique has been used to detect the opening and closing of a single ion channel molecule [48], and rapid progress has been made in understanding the structure and function of ion channels. It was shown that small conformational changes in the channel-forming protein induce a transition from the closed to the open state, allowing up to 10 million ions per second to flow into or out of the cell [49]. All membranes, which are mainly composed of lipids and proteins, are highly dynamic structures in which the membrane environment plays a key role [50]. The lipid regulation of membrane proteins is usually divided in two general mechanisms: (1) direct regulation due to lipid interaction with proteins; (2) the modification of the properties of the membranes (stiffness, stretches, compression, etc.), which in turn affect membrane protein activity [51]. The activity of the ion channels reflects the time series of the ion current flowing from them. The presence of long-term memory in the time series of the ion current means that time series tend to follow their previous states or escape from that; in other words, it means that the open or closed states of the ion channels do not occur randomly. To measure the long-term memory of a time series, the Hurst exponent is used [52]. Here, multifractal detrended fluctuation analysis (MFDFA), as the commonly accepted method to find multifractal properties of a time series, was used. Multifractality, which is a property of complex systems, such as ion channels, has been recently reported in various works [15,16,20]. Although the origin of multifractality is not yet known, at least in the case of ion channels, there are some interesting features shown (reviewed by Silva et al. [14]). For example, Wawrzkiewicz et al. [53] attributed the presence of long-term memory in the channel dynamics to the fluctuations in membrane thickness near the channel’s location. In our research, the patch–clamp recordings from single fast-activating vacuolar (FV) channels were analysed using the multifractal detrended fluctuation analysis (MFDFA) method. In this method, the ion channel behaviour is reflected by the time series of the ion current passing from it. The results presented here show not only the presence of long-term memory (or long-range correlation) in FV channels of vacuolar membrane (tonoplast) but also indicate that the memory properties are scale-dependent, i.e., that are multifractal. Multifractality is a very intriguing feature which is, in complex system science, related to the hierarchical structure of systems [54]. This suggests that ion channel models, such as Markov models [55,56,57] or even fractal modelling [58], should be extended to capture multifractal features. 

Taking into account the fact that plant vacuoles are highly dynamic organelles and are essential for plant cell growth, we began to explore the vacuolar K^+^ channels of red beet taproots, suspecting that these channels are sensitive to auxin. It should be added here that, in contrast to the vacuolar membrane, the effect of auxin on the plasma membrane K+ channels is well established. For example, it has been shown that auxin-induced growth involves K+ uptake through voltage-dependent, inwardly rectifying K+ channels (ZMK1, Zea mays K+ channel 1), the activity of which contributes to water uptake, and consequently, cell expansion [59,60]. Interestingly, it has also been shown that, apart from the posttranslational, auxin-dependent up-regulation of the K+ uptake channels, auxin also regulates the expression of the maize K+ uptake channel gene, ZMK1 [59]. Significantly less is known about the role of the vacuolar K+ channels in the auxin-mediated growth of plant cells. As was shown in the introduction, the K+ current across the tonoplast is primarily mediated by slow-activating (SV) and fast-activating (FV) vacuolar channels. In the first case, we showed that auxin (IAA) stimulated the activity of the SV channels in red beet taproot vacuoles [61]. This effect resulted from faster channel activation and an increased amplitude and the number of opened SV channels. Recently, using the patch–clamp technique, we also showed [13] that IAA regulates the activity of the fast-activating vacuolar (FV) channels, thereby causing changes in the K+ fluxes across the vacuolar membrane. The addition of IAA to the bath solution of red beet vacuoles significantly increased the amplitude of the single FV channel outward currents (from outside to the vacuole) and the number of open channels. However, luminal auxin reduced both the outward and inward FV currents [13].

The main result of the research described here is the demonstration of the multifractal properties of FV channels in red beet taproot vacuoles. The importance of this observation is also emphasized by the fact that in the presence of plant growth hormone, auxin (indole–3–acetic acid, IAA), the multifractal properties of FV channels in red beet taproot vacuoles were also observed. Interestingly, the multifractal properties of the FV channels also depend on the incubation time of the patches in the presence of IAA. For short times (0 min and 5 min), a decrease in the mean width of the singularity spectrum was observed, while for longer times, the mean width increased. The latter observation may indicate a time-dependent effect of auxin on vacuolar membrane structure and function. This effect may be related to the direct interaction of IAA with channel-forming proteins or its indirect effect on channels by changing the membrane fluidity, which is defined by the degree of molecular movement and disorder that make up lipids [62]. In the first case, IAA anions (which dominate with a bath medium of pH 7.5) may directly interact with FV channel gating, while in the second, indirectly, as a result of the interaction of IAA with the lipids of the lipid bilayer. Such a possibility was recently reported by Hąc–Wydro et al. [63]. Our suggestion that auxin may indirectly change FV channel activity is consistent with the hypothesis proposed by Wawrzkiewicz et al. [54], who attributed the presence of long-term memory in the channel dynamics to the fluctuations in membrane thickness near the channel’s location. 

Taking the obtained results into account, it can be suggested that the multifractal properties of fast-activating vacuolar (FV) K^+^ channels, indicating the existence of long-term memory, should be taken into account in terms of the mechanism of the auxin-induced growth of plant cells. It should also be added that the plant plasma membrane K+ channels are probably characterized by similar properties, but so far, there are no data on this in the literature.

## 6. Conclusions

The main result of the research carried out is the demonstration of the multifractal properties of FV channels in red beet taproot vacuoles. The importance of this observation is emphasized by the fact that, in the presence of the biologically active substance, plant growth hormone auxin (IAA), the multifractal properties of FV channels were also observed. Interestingly, these properties depend on the incubation time of the patches in the presence of IAA, which in turn may suggest that auxin changes the physicochemical properties of the vacuolar membrane.

## Figures and Tables

**Table 1:** The mean value and standard deviation of the singularity spectrum width with respect to the external potential.

U(mV)	*<max (Hq) − min(Hq)>*	*σ(max(H_q_) − min(H_q_))*
100	1.135	0.168
80	1.194	0.125
60	1.081	0.075
−60	1.159	0.134
−80	1.218	0.085
−100	1.239	0.067

**Table 2:** The left-hand side final points coordinates of the singularity spectrum curves for the different incubation times in IAA solution and the control bath. U = −100 mV.

Incubation	*α*	*f(α)*
control	1.403	0.637
0 min	1.459	0.619
5 min	1.481	0.649
10 min	1.410	0.418
15 min	1.360	0.447
20 min	1.281	0.291
25 min	1.347	0.441
30 min	1.360	0.415

**Table 3:** The mean value and standard deviation of the singularity spectrum width with respect to the IAA incubation period. U = −100 mV.

Incubation	*<* *max (Hq) − min(Hq)* *>*	*σ(max*(*H_q_*) − *min*(*H_q_))*
control	1.239	0.067
0 min	1.152	0.142
5 min	1.104	0.117
10 min	1.226	0.145
15 min	1.209	0.132
20 min	1.342	0.156
25 min	1.285	0.164
30 min	1.200	0.119

## Data Availability

Data are contained within the article.
